# Resilience of vernacular and modernising dwellings in three climatic zones to climate change

**DOI:** 10.1038/s41598-021-87772-0

**Published:** 2021-04-28

**Authors:** Khadeeja Henna, Aysha Saifudeen, Monto Mani

**Affiliations:** 1 Centre for Sustainable Technologies, Indian Institute of Science, Bangalore, India; 2 Department of Architecture, College of Engineering Trivandrum, Thiruvananthapuram, India

**Keywords:** Energy science and technology, Engineering

## Abstract

Climate change impacts buildings in multiple ways, including extreme weather events and thermal stresses. Rural India comprising 65% of the population is characterised by vernacular dwellings evolved over time to passively regulate and maintain comfortable indoors. Increasing modernization in rural habitations (transitions) evident from the ingress of modern materials and electro-mechanical appliances undermines the ability of building envelopes to passively regulate and maintain comfortable indoors. While such trends are deemed good for the economy, their underlying implications in terms of climate change have not been adequately examined. The current study evaluates the climate-resilience of vernacular dwellings and those undergoing transitions in response to three climate-change scenarios, viz, A1B (rapid economic growth fuelled by balanced use of energy sources), A2 (regionally sensitive economic development) and B1 (structured economic growth and adoption of clean and resource efficient technologies). The study examines dwellings characteristic to three rural settlements representing three major climate zones in India and involves both real-time monitoring and simulation-based investigation. The study is novel in investigating the impact of climate change on indoor thermal comfort in rural dwellings, adopting vernacular and modern materials. The study revealed higher resilience of vernacular dwellings in response to climate change.

## Introduction

Climate change and its effect on human health, economy and environment is one of the most widely researched topics in the twenty-first century. Building and construction industry, the highest contributor to global emissions and climate change, is responsible for 39% of the global energy- and process-related emissions, with 17% from residential sector^1^. However, buildings are also the most vulnerable to climate change, especially in progressive and developing regions. Climate change could involve gradual/abrupt changes in temperatures to unprecedented rains, floods and cyclones. It impacts building thermal comfort, alters operating conditions and energy demands while rendering existing appliances inadequate^2^. This impact is evident as altered comfortable temperature conditions and increased heating/cooling energy demand. In 2018, 22% of the global final energy was consumed by residential buildings^1^, with 40% of energy utilization on space conditioning. This is projected to increase by 40% by 2050 over 2010 levels based on International Energy Agency’s (IEA) 6 ºC (6DS) scenario^3^. The energy consumed by buildings varies depending on the climate, lifestyle and building typology. Per capita energy consumed by buildings in prosperous countries in a cold climate zone like the United States or Canada, could be 5–10 times that of low-income countries in a warm climate zone in Africa or Latin America^4^. In 2018, India’s per capita residential energy consumption was less than 0.6 MWh while that of the US was over 10 MWh^5^. While 66% of India’s population resides in rural areas, only 18% of US population is rural^6^. However, unlike in westernised regions, the disparity in energy consumption between rural and urban India is huge (96 kWh in rural areas, 288 kWh in urban areas as in 2009)^7^. A 4 × increase in embodied energy (EE) and a 40 × increase in operational energy (OE) respectively has been observed with buildings transitioning from vernacular to modern^8^. If rural life in India rapidly transitions towards global urban lifestyles, the ecological stresses (resource extraction and greenhouse gas emissions) could exasperate global efforts to mitigate climate change. The national statistics indicate rapid transition from traditional climate-responsive houses constructed using locally available materials towards an urban-like dwellings adopting industry manufactured, energy-intensive materials, disregarding the local climate. In 2001, clay tiles roofs (32.5%) had given way to concrete (29%) by 2011^9^. A growth of 89% in the use of concrete and 76% increase in metal/asbestos roofing sheets has been witnessed between 2001 and 2011.

While vernacular dwellings have evolved to passively maintain comfortable indoors, modern dwellings inevitably rely on energy-intensive appliances for comfort. Modern buildings tend to be energy-intensive and result in huge emissions through their lifecycle. Residential energy consumption in India in 2014 was 50 times that in 1971^10^. Such increase could cripple global efforts to mitigate climate change. On the other hand, vernacular dwellings could hold key solutions for mitigation and adaptation to climate change, given their capability to withstand wider temperature variations with lower lifecycle energy-resource intensity^11^. This study attempts to assess the thermal performance of vernacular dwellings in three climate zones in India and the effect of material transitions on the thermal performance of these dwellings. The study also investigates the impact of climate change on their performance.

## Literature review

The effect and severity of climate change on thermal comfort in dwellings can be measured as change in heating and/or cooling demand and resulting energy consumption. Severe cold climates are expected to witness reduction in heating demands, while warmer climates are expected to witness considerable increase in cooling demand^12^. The impact of climate change also varies according to building type. Wang and Chen^13^, studied impact of climate change on seven commercial buildings and two residential buildings in different climate zones across the US and found a net increase in energy consumption in warm and moderate climates while a net decrease in energy consumption in colder climates. Karimpour et al.^14^ investigated the impact of different building envelope (insulation and glazing) configurations in mild Australian temperate climate on energy consumption in current and future climates and recommended higher insulation, double glazing and low emittance glass in response to increased cooling energy demand. Huang and Gurney^15^ studied US building stock, focusing on commercial building types, across three building age classes- pre-1980, post-1980 and new-2004, to understand their performance under climate change. The buildings equipped with the newest technology with more energy efficient equipment and better insulation exhibited higher efficiency in maintaining comfortable indoor environment.

Naturally ventilated (NV) vernacular dwellings constructed using local materials have lower embodied energy and ecological footprint. Mani et al.^16^ estimated the ecological footprint of conventional dwellings to be 2.5 times that of traditional dwellings in West Bengal, India. Vernacular buildings also tend to be resilient in wider range of temperatures in providing comfort when compared to conventional buildings^17–19^. Dili et al.^20^ studied vernacular and conventional dwellings in Kerala, India, with warm-humid climate, and confirmed that vernacular dwellings outperformed conventional dwellings in maintaining indoor comfort across seasons. Shastry et al.^21^ studied the impact of modern transitions on rural dwellings in West Bengal, India, and found an increase in average indoor temperatures from 7 to 10 °C. Given the lower EE and OE energy and higher resilience to maintain indoor thermal comfort in response to wider variations in weather conditions, vernacular dwellings could hold important insights for mitigation and adaptation to climate change.

## Methodology

India is broadly classified into five climatic zones- hot-dry, warm-humid, composite, temperate and cold^22^. In this study, three distinct villages belonging to warm-humid, temperate and cold climate zones have been selected to represent warm, moderate and cold climatic conditions in India, respectively. A typical vernacular dwelling from each village was selected for detailed investigation into climate-resilience. Real-time indoor air temperatures at different locations in the dwellings were recorded at 30-min intervals using calibrated Resistance Temperature Detector (RTD) data loggers (0.05 °C resolution and ± 1 °C accuracy). Real-time measurement of indoor parameters proceeded according to Class III level of detail as defined in^23^, widely adopted in thermal comfort field study research. It was important to prevent the loggers from interfering with the daily lives of the inhabitants as these loggers were being installed in occupied houses for almost a year. Calibrated simulation models of the vernacular dwellings were developed using DesignBuilder (v 3.4), an integrated building performance simulation package^24^. The model calibration essentially involved a correlation with real-time climatic-performance data (see Sect. 4). In order to examine performance of vernacular dwellings in response to climate change, weather files for both typical and future climate scenarios were generated using *Meteonorm*. *Meteonorm* is a global climatological tool, that derives data from weather stations between 1991 and 2010 and integrates IPCC AR4 (Intergovernmental Panel on Climate Change- Fourth Assessment Report) emission scenarios^25^. Three future scenarios, namely, A1B (rapid economic growth fuelled by balanced fossil/non-fossil energy use), A2 (regionally oriented economic development characterised by less innovation) and B1 (structured economic growth and adoption of clean and resource efficient technologies) are explored in this paper. Both A1B and A2 are high emission scenarios, with A2 resulting in higher global surface warming in the second half of the twenty-first century. B1 on the other hand is one of the scenarios with lowest emissions and global surface warming^26^. Modern transitions in each of the habitations were studied based on trends revealed through satellite imagery, field visits and government reports, and were incorporated in the simulation models to examine the impact of future climate change scenarios.

**Figure 1:**
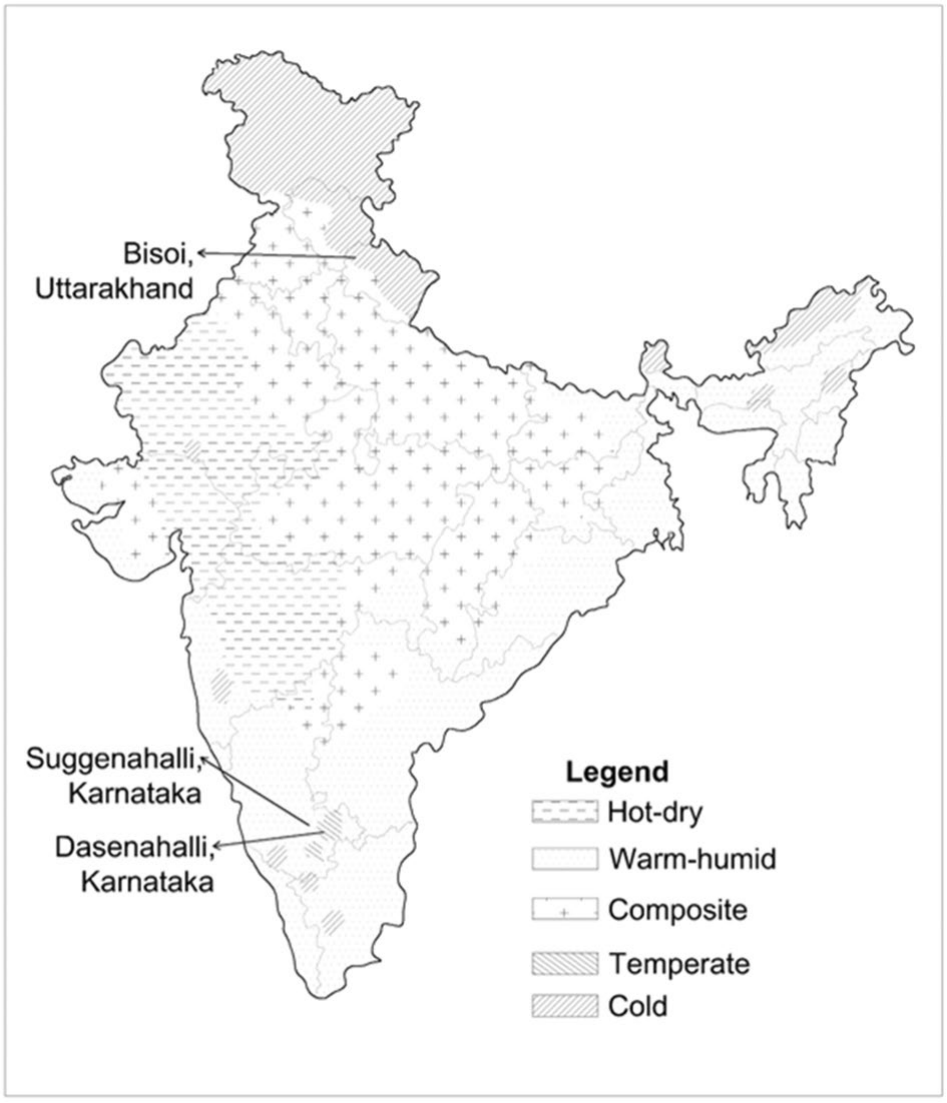
Climatic zones in India. (Adapted from National Building Code of India, 2005^22^) and selected rural settlements: Figure shows the location of the rural settlements studied in this paper on the climate zone map of India. Suggenahalli lies in Warm-humid zone, Dasenahalli in Temperate zone and Bisoi in Cold zone. The authors used Autodesk AutoCAD 2018 (Product version: O.49.0.0 AutoCAD 2018) https://www.autodesk.com/products/autocad/overview?support=ADVANCED&plc=ACDIST&term=1-YEAR&quantity=1 to draw the map and mark the rural settlements on it.

This study comprises three agrarian rural settlements (Fig. 1) namely Suggenahalli, Karnataka (12.816° N, 76.993° E), Dasenahalli, Karnataka (13.146° N, 77.465° E) and Bisoi, Uttarakhand (30.971° N, 77.928° E) belonging to warm-humid, temperate and cold climate zones respectively. In addition to the willingness of the inhabitants, these settlements were identified for unique vernacular architecture (Table 1) and an evident trend of modern transitions. The vernacular dwellings in these villages are naturally ventilated and constructed using locally available materials with local traditional know-how passed over generations.

**Table 1:** Details of the vernacular dwellings in the three settlements.

	Form	Roof	Walls	Flooring	Fenestration
Suggenahalli	Single-storeyed, rectangular plan with central courtyard	Sloping roof, Mangalore tiles on timber battens and large overhangs; Flat Maalige roof (mud roof supported by timber planks)	Thick rubble walls with mud plastering indoors	Mud flooring	Fewer windows with timber frame and shutter
Dasenahalli	Single-storeyed, rectangular plan	Sloping clay tile roof on bamboo truss; large roof overhangs	Thick mud-block walls with lime plastering	Mud flooring	Small and fewer windows with timber frame and shutters
Bisoi	2 storeys, rectangular plan, with projected upper floor	Sloping roof with slate laid over timber battens	Ground floor walls—Stone masonry with intermediate timber ties; First floor walls—timber panels	Ground floor mud flooring; timber flooring in first floor	Very small and windows with timber shutters

The proximity and improved connectivity of Suggenahalli and Dasenahalli to Bengaluru city and Bisoi to Dehradun has helped spur modern lifestyles. Access to electricity and modern construction materials is evident as transitions in the dwellings. These transitions that mimic urban habitations are characteristic to many rural habitation and involve traditional local materials giving way to energy-intensive exotic materials^8,27,28^.

**Figure 2:**
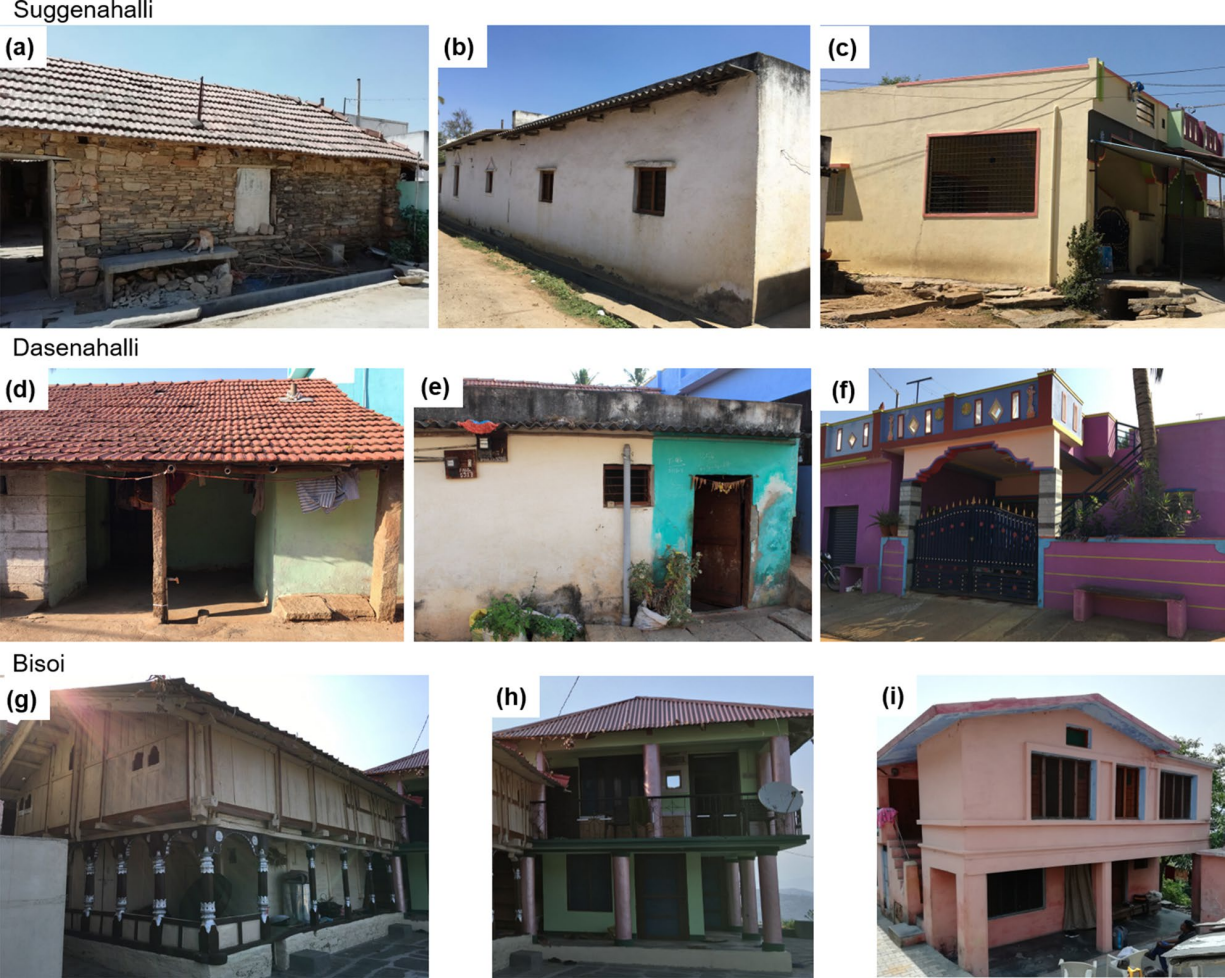
Stages of material transition (from left to right) in vernacular dwellings in the three villages: Stages of transition in the use of construction materials for each rural settlement is shown in the figure. Images in each row from left to right shows transition from traditional materials to conventional materials. (**a**), (**d**) and (**g**) represents the typical vernacular construction using traditional materials in Suggenahalli, Dasenahalli and Bisoi, respectively. (**b**), (**e**) and (**h**) represent a common intermediate stage in the process of transition using conventional but mostly temporary materials in the three villages. (**c**), (**f**) and (**i**) are the final products of transitions in the three villages using conventional factory-made materials which are more permanent in nature.

Transitions in vernacular dwellings were characterised by progressive inclusion of modern materials, while retaining the original form. Newer constructions adopted modern construction materials, seldom carrying a semblance of traditional form and indoor spaces. In Suggenahalli (Fig. 2a–c), the thick rubble walls were increasingly being replaced by slender brick masonry, the pitched clay-tile roofs were replaced by flat AC sheet roofing and then by reinforced cement concrete (RCC) slabs, and the cool earthen floors have made way to cement flooring. In Dasenahalli (Fig. 2d–f), clay from the agricultural fields were moulded into mud blocks to construct walls. These walls were increasingly being replaced by cement blocks, pitched clay tiled roof by tin/AC sheets and ultimately by flat RCC roof and the mud flooring by cement and tiles. Bisoi, which once depended on forest-harvested timber for houses, is faced with state regulations restricting the use of forest produce. This also has forced villagers to look for alternate building materials, with the older generation preferring vernacular dwellings, and the younger city-educated/employed generation preferring conventional dwellings. The geographic isolation by mountain ranges has moderated the rate of transitions and helped in higher retention of vernacular dwellings. The transitions in Bisoi (Fig. 2g–i) mostly involve replacing of timber walls on the first floor by brick walls while retaining the ground floor stone and timber wall construction, the slate roof by metal sheets and pitched RCC roof and mud/timber flooring by cement flooring. Flat RCC roofs originally constructed revealed cracks under heavy snow load and were eventually replaced by sloping RCC roof. In all the three villages, material transitions remained the most prominent and evident type of transition. The current study examines the primary material transition in vernacular dwellings retaining their form and orientation for their thermal performance and resilience to climate change. Table 2 summarises the vernacular and conventional building materials used in constructing the simulation models for the three case studies.

**Table 2:** Details of envelope materials of vernacular and conventional dwelling models.

Location	Building element	Vernacular dwelling		Conventional dwelling	
		Construction	U value (W/m^2^ K)	Construction	U value (W/m^2^ K)
Suggenahalli	Wall	450 mm random rubble wall with mud plaster inside	3.91	230 mm Brick masonry with 12 mm cement plaster	2.24
	Roof	20 mm thick Mangalore Tiles on timber rafters	6.14	100 mm RCC with 2% reinforcement	4.79
		300 mm Maalige Roof	2.06		
	Floor	Earth flooring	1.72	Cement flooring	1.57
	Windows	20 mm Timber panelled shutter	2.22	Single glazed window	5.78
Dasenahalli	Wall	450 mm mud wall with clay plaster	1.33	200 mm concrete block masonry with 12 mm cement plaster	3.07
	Roof	Pitched clay tiles on Timber battens	2.33	Flat RCC slab	5.32
	Floor	Mud Flooring	2.06	Cast concrete with Ceramic tiling	2.59
	Windows	Timber shutter	0.64	Single pane glass	3.84
Bisoi	Wall (GF)	550 mm stone and timber wall	1.78	550 mm stone and timber wall	1.78
	Wall (1st F)	Panelled timber wall	1.23	Brick Wall	2.79
	Roof	Slate tiles over timber battens	1.95	Pitched RCC roof	5.56
	Floor (GF)	Stone flooring	2.91	Cement mortar over stone	2.81
	Floor (1st F)	Timber flooring with woollen carpet	0.81	RCC Slab	4.32
	Windows	Wood panel	0.64	Single pane glass	3.84

**Figure 3:**
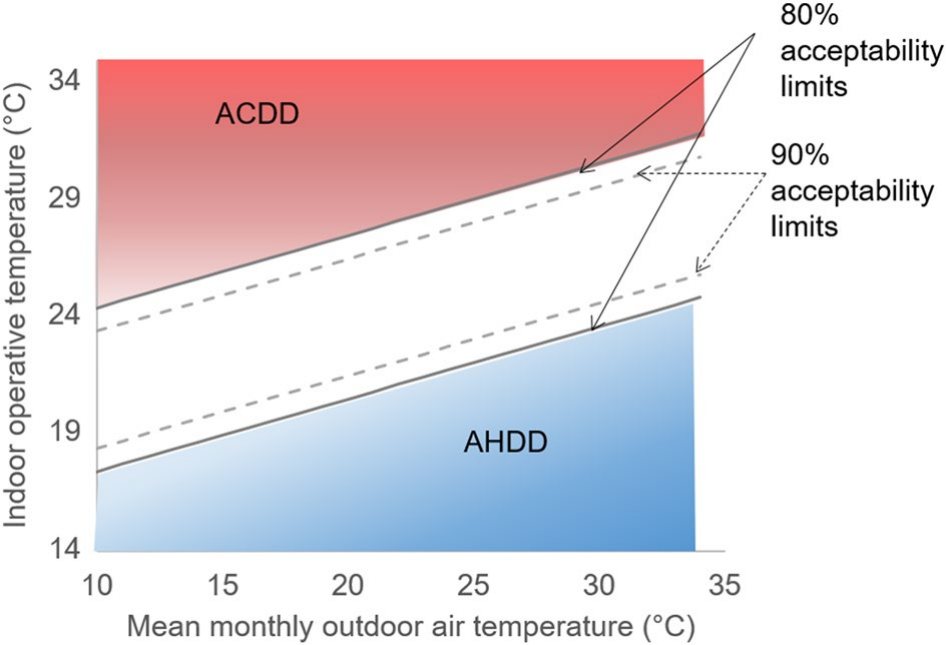
Representation of ACDD and AHDD based on Adaptive Thermal comfort model, ASHRAE 55: The plot shown in the figure represents the Adaptive thermal comfort model defined in ASHRAE 55. Solid line indicates 80% acceptability limits and dotted line indicates 90% acceptability limits. Figure depicts the method adopted for calculating Adaptive Heating Degree Days (AHDD) and Adaptive Cooling Degree Days (ACDD). Indoor operative temperatures falling above or below the 80% acceptability limits multiplied by the duration for which the temperature lasts are used to calculate ACDD and AHDD, respectively. Duration for which the indoor operative temperatures fall above the 80% acceptability limit will require active cooling to attain comfort. Similarly, duration for which the indoor operative temperatures fall below the 80% acceptability limit will require active heating for comfort.

Studies focusing on naturally ventilated buildings rely on adaptive thermal comfort models to assess building climatic performance. These models more precisely accommodate the natural physiological ability to adapt indoor comfort requirements in response to external climatic (temperature) conditions. The performance of dwellings in this study adopts Adaptive Heating and Cooling Degree Days (AHDD and ACDD) calculated based on the Adaptive thermal comfort model (ATCM) incorporated in the ASHRAE 55 standard, 2010^29^. ATCM takes into account the acceptability of wider range of temperatures by occupants in naturally ventilated buildings, especially in tropical climates^30^. It illustrates a linear dependence of indoor operative temperatures on mean monthly outdoor temperatures and also describes a 90% and 80% acceptability limits indicating the percentage of occupants expressing comfort^31^. The days when indoor temperatures exceed acceptability limits, the corresponding ACDD and AHDD are computed as a measure of the augmented cooling or heating need (see Fig. 3):

1 $${\mathbf{Annual}}{ }{\mathbf{ACDD}} = { }\mathop \sum \limits_{{{\mathbf{i}} = 1}}^{365} ({\mathbf{T}}_{{{\mathbf{op}}.{\mathbf{i}}}} - {\text{T}}_{{{\text{b}}.{\text{m}}}}^{{{\text{ul}}}} )\quad {\mathbf{when}}\quad {\mathbf{T}}_{{{\mathbf{op}}.{\mathbf{i}}}} > {\text{T}}_{{{\text{b}}.{\text{m}}}}^{{{\text{ul}}}}$$

2 $${\mathbf{Annual}}{ }{\mathbf{AHDD}} = { }\mathop \sum \limits_{{{\mathbf{i}} = 1}}^{365} ({\text{T}}_{{{\text{b}}.{\text{m}}}}^{{{\text{ll}}}} - {\mathbf{T}}_{{{\mathbf{op}}.{\mathbf{i}}}} )\quad {\mathbf{when}}\quad {\mathbf{T}}_{{{\mathbf{op}}.{\mathbf{i}}}} < {\text{T}}_{{{\text{b}}.{\text{m}}}}^{{{\text{ll}}}}$$

where $T_{op.i}$ is the daily operative temperatures and $T_{b.m}^{ul}$ and $T_{b.m}^{ll}$ are the upper and lower limits of the monthly base temperature above and below which cooling and heating requirements surface. $T_{b.m}^{ul}$ and $T_{b.m}^{ll}$ are calculated based on ATCM for each month using the relation:

3 $${\text{T}}_{{{\text{b}}.{\text{m}}}}^{{{\text{ul}}}} = 0.31 \times {\mathbf{T}}_{{{\mathbf{mm}}}} + 17.8 + {\mathbf{x}}$$

4 $${\text{T}}_{{{\text{b}}.{\text{m}}}}^{{{\text{ll}}}} = 0.31 \times {\mathbf{T}}_{{{\mathbf{mm}}}} + 17.8 - {\mathbf{x}}$$

where *T*_*mm*_ is the mean monthly outdoor temperature and **x** is the variability based on acceptability limit, which is 3.5 for 80% acceptability and 2.5 for 90% acceptability^31,32^.

## Model calibration

**Figure 4:**
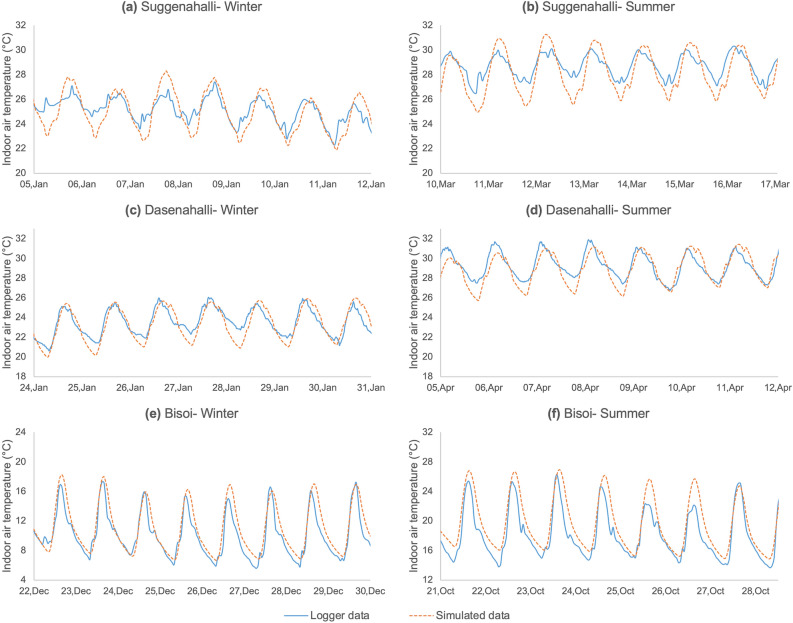
Comparison of simulated and measured temperature data for a representative week-long period in Summer and Winter for the dwellings studied in the three settlements: Figure shows comparison of measured and simulated hourly indoor air temperatures (in °C) for a representative week in winter and summer for the three dwellings to understand how the simulation model has been able to represent the actual indoor operating conditions prevailing in the dwellings. Solid blue line shows the real-time temperature measurements recorded by the loggers, while the dotted orange line shows the simulated data. The simulation models tend to closely imitate real-time operating conditions, suggesting that the models are representative enough to be used for studying the behaviour of the original dwellings.

**Figure 5:**
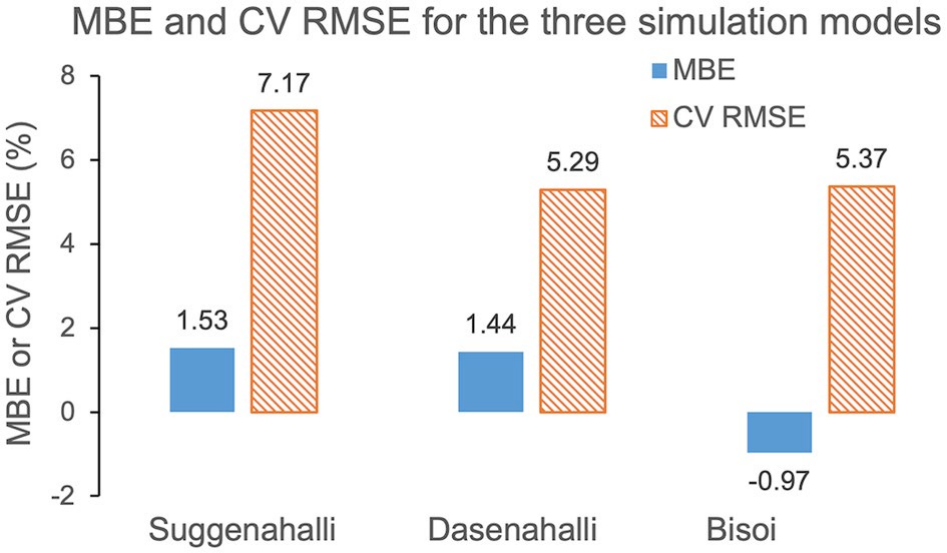
MBE and CV RMSE between measured and simulated temperature data for the dwellings in the three settlements: Figure shows the Mean Bias Error (MBE) and Coefficient of Variation of Root Mean Square Error (CV RMSE) in percentage calculated between the measured and simulated indoor air temperature data for the dwellings studied in each of the three settlements. The blue coloured solid bar shows MBE, while orange coloured bar with diagonal stripes shows CV RMSE. Both MBE and CV RMSE for all the three settlements are within the limits prescribed by ASHRAE 14 guideline.

ASHRAE Guideline 14 suggest use of Mean Bias Error (MBE) and Coefficient of Variation of Root Mean Square Error (CV RMSE) for calibration of simulation model based on measured performance data^33–35^. MBE and CV RMSE indicate the relative and accumulated divergences between measured and simulated value, respectively. According to the guideline, for an acceptable calibrated simulation model, MBE hourly data should lie within ± 10% and CV RMSE hourly data should not exceed 30%. For this study, the simulated indoor air temperatures were calibrated based on real time measurements. Royapoor and Roskilly^36^ in their study on performance of an office building had validated their EnergyPlus virtual building model by calculating MBE and CV RMSE. For better reliability, ASHRAE recommends using both statistical and graphical approaches to model calibration^33,37^, which has been adopted in this study: firstly, by graphically comparing the measured and simulated indoor air temperatures, and secondly, by calculating the MBE and CV RMSE. Figure 4 illustrates the concurrence between measured and simulated weeklong summer and winter temperatures for the three case studies. Since summer data for Bisoi was not accessible, a warm week in October was relied on as representative for summer. A one-on-one match between real-time and simulated temperature is rarely feasible given the variability in the external climatic data between the simulation model and on-site conditions. Also, variations attributed to non-routine indoor occupancy are difficult to precisely predict and include in the simulation model^38,39^. This approach to calibration has been adopted for reliable building performance studies^36,37,39,40^. Figure 5 illustrates the MBE and CV RMSE for the three simulation models being well within recommended favourable limits for calibration. Moreover, the lower error values indicate improved reliability of the simulation results^33,35^. MBE and CV RMSE between measured and simulation data are calculated using Eqs. (5) and (6):

5 $$ {\mathbf{MBE}}{ }\left( {\text{\% }} \right) = { }\frac{{\mathop \sum \nolimits_{{{\mathbf{i}} = 1}}^{{\mathbf{N}}} \left( {{\mathbf{m}}_{{\mathbf{i}}} - {\mathbf{s}}_{{\mathbf{i}}} } \right)}}{{\mathop \sum \nolimits_{{{\mathbf{i}} = 1}}^{{\mathbf{N}}} \left( {{\mathbf{m}}_{{\mathbf{i}}} } \right)}} $$

6 $$ {\mathbf{CV}}{ }{\mathbf{RMSE}}{ }\left( {\text{\% }} \right) = { }\frac{{\sqrt {\left( {\mathop \sum \nolimits_{{{\mathbf{i}} = 1}}^{{\mathbf{N}}} \left( {{\mathbf{m}}_{{\mathbf{i}}} - {\mathbf{s}}_{{\mathbf{i}}} } \right)^{2} /{\mathbf{N}}} \right)} }}{{{\overline{\mathbf{m}}}}} $$

where *m*_*i*_ and *s*_*i*_ are measured and simulated data points for *i*th hour, $\overline{m}$ is the average of measured data points and *N* is the total number of data points.

## Results

**Figure 6:**
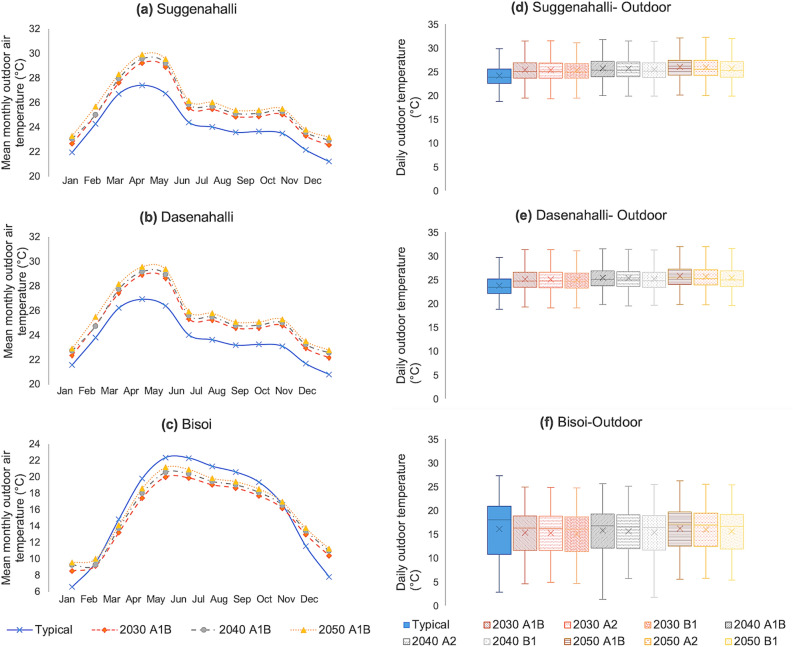
(**a**)–(**c**) Mean monthly outdoor air temperatures for typical and A1B scenario for 2030, 2040 and 2050 and (**d**), (**e**) the range of daily outdoor air temperatures for the three settlements for typical scenario and all future scenarios: (**a**), (**b**) and (**c**) shows the mean monthly outdoor air temperature (in °C) for typical scenario and A1B scenario for the years 2030, 2040 and 2050 for the rural settlements Suggenahalli, Dasenahalli and Bisoi, respectively. The same is not shown for A2 and B1 scenarios but they show similar trend with increasing temperatures with each passing decade. (**d**), (**e**) and (**f**) show the range of outdoor air temperatures (in °C) for typical and all three scenarios for future years 2030, 2040 and 2050 for the rural settlements Suggenahalli, Dasenahalli and Bisoi, respectively. The temperatures tend to increase with each decade with the highest increase in A1B scenario and lowest in B1 scenario for both Suggenahalli and Dasenahalli. In case of Bisoi winters tend to get warmer than typical conditions while summers tend to get cooler than typical conditions. The decadal increase in temperature can be witnessed here as well.

With regards to climate change scenarios, for both Dasenahalli and Suggenahalli, the likely outdoor temperatures are much higher than the prevalent trends for all the three scenarios, with a steady decadal increase (see Fig. 6a, b, d and e). A1B witnesses the highest increase in temperature across the years while B1 had the lowest. Notably, increase in summer (March–May) temperatures are higher than increase in winter (December–February) temperatures. In Bisoi (Fig. 6c and f), the prevalent mean monthly temperatures ranging from 6 to 22 °C narrowed to 8–21 °C in the future years, while also registering a decadal increase. Here, while future winter temperatures are likely to increase, summer temperatures are likely to decrease.

**Figure 7:**
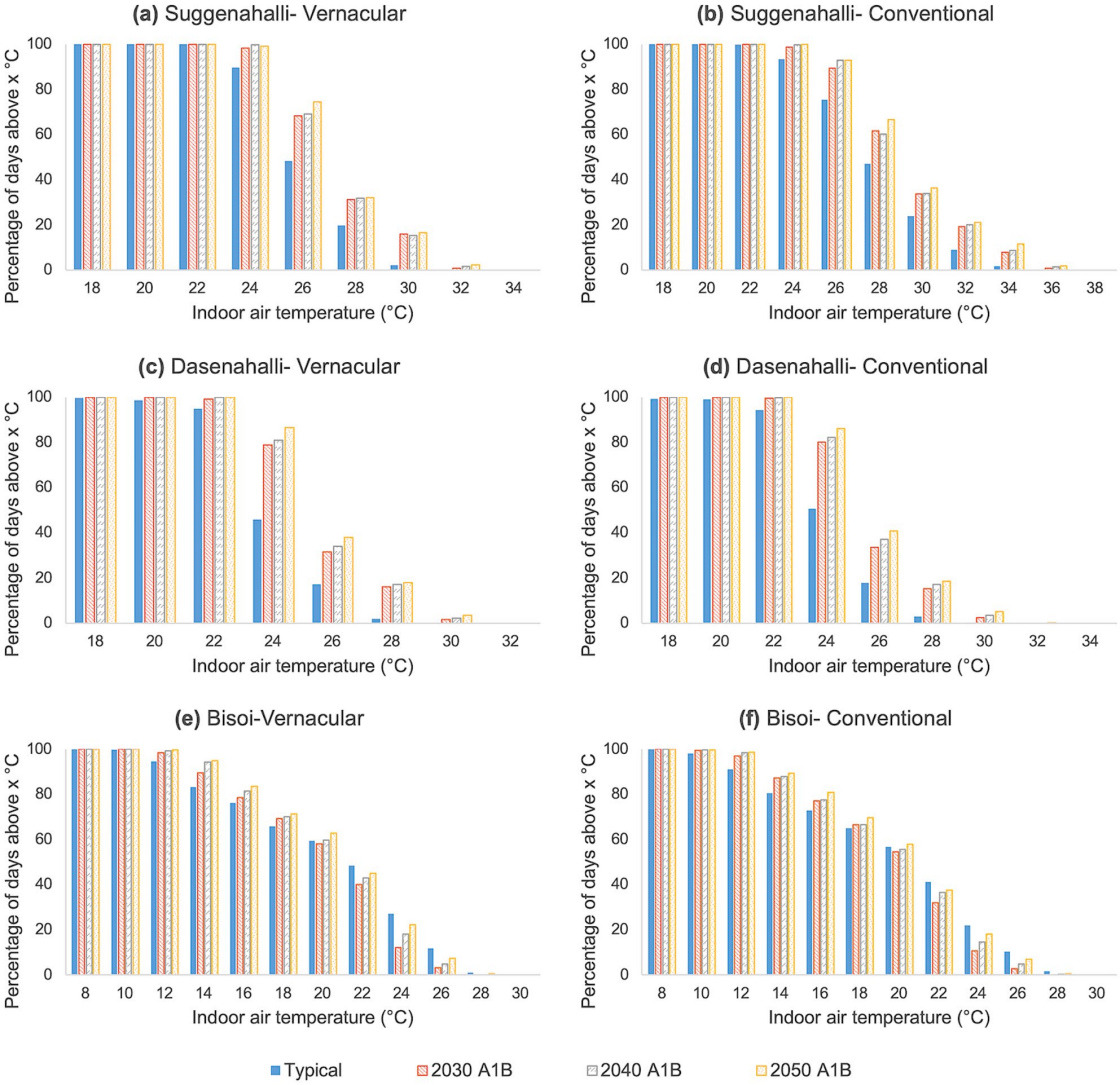
Percentage of days above a daily temperature given on abscissa for vernacular and conventional dwelling in the three settlements for typical scenario and future years under A1B scenario: Each bar in the plot shows the percentage of days in a year for which the mean daily indoor air temperature (in °C) is greater than the corresponding temperature (in °C) given on the abscissa. The plot helps to understand the range of temperatures and extent to which those temperatures prevail inside a dwelling throughout the year. This is shown for typical scenario and future years for A1B scenario. (**a**) and (**b**) shows the same for vernacular and conventional dwelling in Suggenahalli, (**c**) and (**d**) for dwellings in Dasenahalli and (**e**) and (**f**) for dwellings in Bisoi. Higher temperatures tend to persist in the dwellings in future years compared to typical conditions especially for conventional dwelling in case of both Suggenahalli and Dasenahalli. This is more evident in Suggenahalli than Dasenahalli. In the case of Bisoi, the frequency of lower temperatures in higher in the future compared to typical conditions, while frequency of higher temperature is higher in typical conditions than future years.

Figure 7 shows daily indoor temperature distribution in the vernacular and conventional dwelling in both typical and future A1B climate scenario for the three cases. The graphs reveal the % days/year with an average daily temperature greater than the value on the abscissa. In the vernacular dwelling in Suggenahalli (Fig. 7a), climate change tends to increase indoor temperatures in the future. When the indoor temperatures exceeded 30 °C for only 2% of days in a normal year, in 2030, 16% of the days revealed temperature above 30 °C. A similar trend can also be noted in the conventional dwelling (Fig. 7b). In typical climate the indoor temperatures in conventional dwelling exceeded 30 °C for 24% of the days and 34 °C for 2% of the days. By 2030, indoor temperatures exceeded 30 °C for 34% of the days and 34 °C for 8% of the days. The persistence of higher temperatures in conventional dwelling shows the effect of material transitions. Dasenahalli (Fig. 7c and d) also shows a similar trend, though the difference between vernacular and conventional dwelling is not as high. In typical climate, average daily indoor temperatures exceed 26 °C, 17% of the days in vernacular dwelling and 18% in conventional dwelling. Climate change does influence the thermal environment in the dwellings and tends to increase the daily indoor temperatures as is evident from the figure. The temperature in both the dwellings does not exceed 32 °C in any case. Bisoi (Fig. 7e and f) recorded a wider temperature range from 8 to 28 °C. Even though there is a general increase in temperatures due to climate change, at higher temperatures the trend reverses. Future years does not witness an increase in higher temperatures compared to typical climate. The daily indoor temperature in both the dwellings does not exceed 28 °C. Bisoi being in cold climate zone, the concern should be on the persistence of lower temperatures. In vernacular dwelling, temperature falls below 16 °C, 24% of the time in typical climate while in 2030 A1B scenario it falls below 16 °C for 22% of the time. While in conventional dwelling, temperature falls below 16 °C, 13% of the time in typical climate while in 2030 A1B scenario it falls below 16 °C for 23% of the time. Transitions and climate change seem to maintain comfortable temperature range indoors.

**Figure 8:**
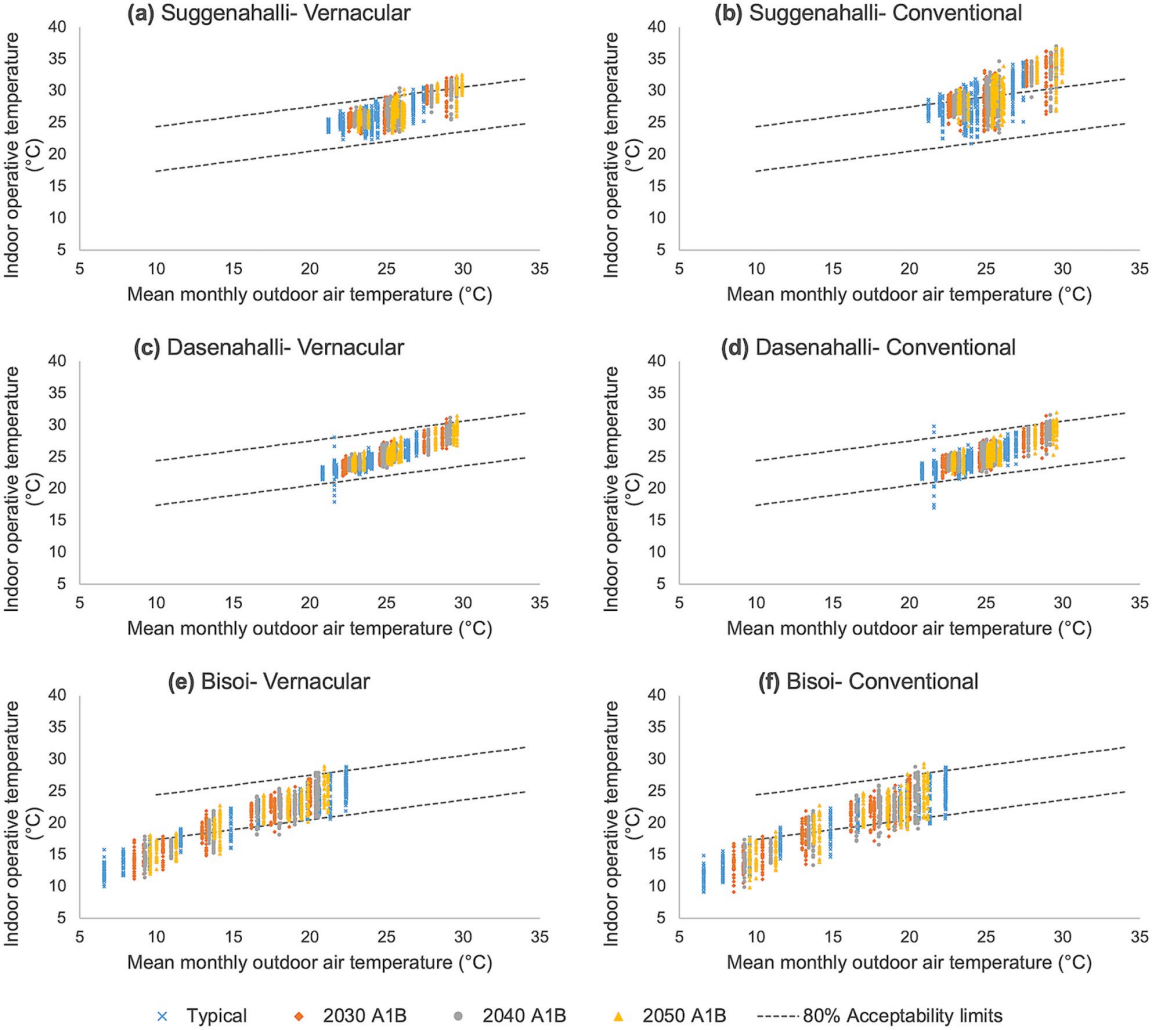
Indoor operative temperature against mean monthly outdoor air temperature for vernacular and conventional dwellings in the three settlements in typical and future climate for A1B scenario: Each plot shows the daily indoor operative temperatures (in °C) inside the dwelling throughout a year plotted against mean outdoor air temperature (in °C) for the corresponding month. The two dotted lines shown in each figure indicates the 80% acceptability limits prescribed by ASHRAE 55 Adaptive thermal comfort model. The points lying within the 80% acceptability limit band are considered to be comfortable by at least 80% of the inhabitants, while those lying outside indicate thermal discomfort. Higher number of points lying outside the band indicate higher discomfort in the given dwelling. Comparing (**a**) and (**b**), conventional dwelling tends to be highly uncomfortable throughout the year for typical as well as future scenarios. Comparing (**c**) and (**d**), there is only marginal difference between vernacular and conventional dwelling in Dasenahalli. Comparing (**e**) and (**f**), though both vernacular and conventional dwelling tend to be uncomfortable at lower outdoor temperatures, vernacular dwelling seems to be warmer than conventional dwelling providing better comfort in cold weather.

In Fig. 8, the daily operative temperatures for each dwelling in the three villages are plotted against mean monthly daily temperatures for typical climate and future years for A1B scenario. It also shows the 80% acceptability limits as prescribed by ASHRAE 55. This figure forms the basis for the calculation of ACDD and AHDD. The vernacular dwelling in Suggenahalli (Fig. 8a) maintains an indoor operative temperature within the acceptability limits for most part of the year for typical climate but exceeds the upper limit for future years at higher outdoor temperatures. On the other hand, the conventional dwelling (Fig. 8b) fails to maintain indoor temperatures within the acceptability limits for both typical as well as future years for large part of the year. This shows that transitions hugely impact the indoor operative conditions in the dwelling. In the case of Dasenahalli (Fig. 8c and d), the indoor operative temperatures are maintained within the acceptability limits for both vernacular and conventional dwellings for typical climate and future years. In Bisoi (Fig. 8e and f), for both the dwellings, the temperatures fall below the lower acceptability limit for a large part of the year for typical climate and future years.

**Figure 9:**
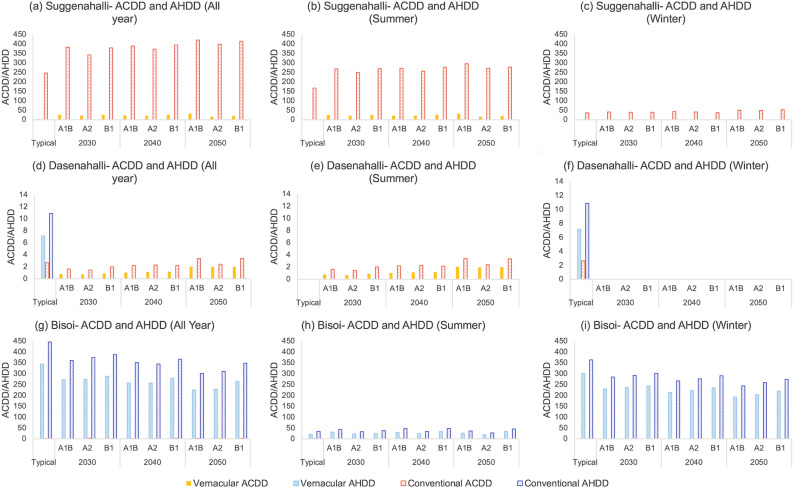
All year, summer and winter AHDD and ACDD for the dwellings in the three villages for typical and future climate scenarios: Each plot depicts the Adaptive heating degree days (AHDD) and Adaptive cooling degree days (ACDD) calculated for vernacular and conventional dwelling for typical scenario and future years 2030, 2040 and 2050 for A1B, A2 and B1 scenarios. Plots from left to right shows typical and future ACDD and AHDD calculated for the entire year, for summer and winter for each rural settlement for both vernacular and conventional dwelling. From (**a**), (**b**) and (**c**) conventional dwelling in Suggenahalli tend to demand active cooling for most part of the year compared to vernacular dwelling. In case of Dasenahalli (**d**), (**e**) and (**f**), it can be seen that demand for heating or cooling is fairly low, cooling demand is slightly higher in conventional dwelling. From (**g**), (**h**) and (**i**), heating demand is high in both dwellings in Bisoi, especially in winter, with heating demand higher in conventional dwelling than vernacular dwelling.

As followed in the comparative evaluation of vernacular buildings under climate change, AHDD and ACDD were computed for the material transitions in dwellings under typical and future (A1B, A2 and B1) climate scenarios. The dwelling in Suggenahalli (Fig. 9a–c), being in a warm-humid climate zone, do not require any heating throughout the year for all scenarios alike. For the vernacular dwelling, ACDD tend to be quite low in typical climate with small increase in future years. Both climate change and transitions tend to have a serious effect on thermal comfort inside the dwelling in warm-humid climate zone. When the building materials changed from vernacular to modern, ACDD increased from 4 to 246 in typical climate condition (4 to 167 in summer and 0 to 36 in winter). This high difference in degree days between vernacular and conventional dwelling is evident from Fig. 8a and b shows that a large number of data points lie above the acceptability limit in conventional dwelling compared to vernacular dwelling. Climate change further increased ACDD in the future years with highest increase in A1B scenario and lowest in A2 scenario. Cooling requirement increases considerably due to climate change and transitions especially in summer. In the case of Dasenahalli (Fig. 9d–f), both AHDD and ACDD are very low in both vernacular and conventional dwelling in typical climate. ACDD is zero in all future years while AHDD, though small, steadily increases in future years, especially for A1B scenario. Transition also affects the thermal comfort in the dwellings where transitions increased both ACDD and AHDD in typical climate, marking an increase in cooling as well as heating demand. In all future years across scenarios, transitions increase ACDD in the dwelling. In Dasenahalli, ACDD and AHDD are not severe enough to require cooling or heating appliances. Bisoi (Fig. 9g–i) being in cold climate zone, does not require cooling throughout the year for both typical as well as future climatic conditions. The warming climate in Bisoi tend to decrease heating demand in the dwelling in both vernacular and conventional dwelling. Transitions have an adverse effect on the thermal comfort in the dwelling as it increases heating demand in the dwelling by an average of 25% compared to the vernacular dwelling in winter. This shows the effectiveness of insulation provided by the timber walls and the timber and slate roofing in vernacular dwelling over conventional materials.

## Discussion

The study investigated the effect of climate change and material transitions (replacing local traditional materials with conventional materials) on vernacular dwellings in three villages in India across three different climate zones. The impact of climate change on the dwellings were examined for the years 2030, 2040 and 2050 under the IPCC SRES scenarios A1B, A2 and B1 and compared with typical climate which is an average of 20 years of recorded weather data. In both Suggenahalli and Dasenahalli, climate change increased the outdoor temperatures throughout the year affecting the indoor environment as well. The effect of climate change was more pronounced in the case of Suggenahalli located in warm-humid climate zone, making the indoors warmer than the typical, climate demanding the use of cooling appliances. Due to climate change, winter outdoor temperatures increased while summer temperatures decreased in Bisoi. Summer temperatures can be expected to increase further in the second half of the century. Climate change has helped increase indoor temperatures which reduced heating demand in the dwellings. In warm climates, A1B scenario had the worst impact on the weather and the indoor temperatures, while B1 scenario which was a low emission scenario had comparatively lower impact on the temperatures. On the other hand, in cold climates A1B helped reduce the heating demand much better than A2 and B1 scenarios.

For both Suggenahalli and Dasenahalli, the vernacular dwelling maintained comfortable indoor operating conditions for most part of the year. The conventional dwelling in Dasenahalli also was able to maintain comfortable indoor conditions. Transitions in Suggenahalli led to warmer indoor environment pushing it beyond the acceptability limits. In case of Bisoi, indoor temperatures fall below acceptability limit for both vernacular and conventional dwelling. The thermal insulation provided by the traditional materials in the vernacular dwelling helped it to perform better than the conventional dwelling in maintaining warmer indoors. Vernacular dwellings seem to perform better than the conventional dwellings in all the three cases for typical as well as future climate. Conventional dwelling that replaced the traditional materials with modern materials failed to adequately respond to changes in climate, compromising indoor thermal comfort and necessitating dependence on active space conditioning.

Modern material transitions in dwellings compromised the thermal comfort in the dwellings across all climate zones, though the effect was not severe in Temperate climate as it was in Warm-humid and  Cold climates. Climate change further exasperated the thermal comfort in dwellings in warm climates but seemed favourable in cold climates. The study helps to verify the climate-resilience of vernacular dwellings to perform in the context of much imminent climate change and shows that modern dwellings constructed using modern industry manufactured materials, to meet the modern aspirations of inhabitants may not perform in the event of climate change. This confirms the need for reinterpretation in design of houses that meet the modern aspirations of the people and perform in the future climate scenarios. The study indicates that vernacular dwellings hold key answers in the direction of mitigation and adaptation strategies in response to climate change and advocates designers to understand the present and future climatic conditions and future demands in the local context, to appreciate and scientifically validate traditional wisdom while designing buildings for the future. Designers must ensure that the dwellings they construct not only meet the aspirations of the modern inhabitant but also perform under future climatic pressures.

## Limitations of the study and scope for future work

i. A country like India is home to diverse vernacular architecture which varies with climate, culture, customs, and available resources. This paper studies three rural settlements with unique vernacular architecture and evident transitions in three different climate zones in India. The authors have selected the settlements such that the vernacular architecture is representative of the climate zone they are in. Inclusion of more case studies and other climate zones would increase the scope of the paper and present a better estimate on the nature of transitions and their ramifications on resource and energy requirement and vulnerabilities to climate change. The current study is a step in this direction and is also a methodological contribution for such studies, tested for three diverse settlements. The selection of settlements for the study was difficult as it required a combination of both vernacular and modern dwellings and those that are in transition. Overcoming scepticism of villagers and gaining their trust was an important requirement to ensure the corporation of the villagers as the study extended for more than a year in each of the villages. Extending this study to cover the diversity of habitations in India, would require networking with local academic institutions, a larger group and a longer time to identify appropriate interventions/mitigation measures in response to climate change.
ii. Understanding the contribution of each stage of transition on the thermal performance of the dwelling is also important to identify critical stages of transition and adopt preventive or adaptive strategies to improve thermal comfort in the dwelling without compromising the requirements of the inhabitants. Future work will involve investigation of individual stages of transitions and their effects on thermal comfort in the dwelling to assess the contribution of each transition on the performance of the dwellings. The study could subsequently evolve into regional building codes and recommendations for interventions in response to climate change, and the revalidation of vernacular building typologies for their relevance in the modern world. Further, the study could promote dependence on local decentralized circular economies that rely on local resources and skill, thereby lowering the global carbon footprint.
iii. The current study is limited to understanding the thermal performance of the dwellings to changing climate. Studies have reported increased discomfort among occupants due to transitions^20,21^ as well as climate change^2^. The response of the occupants to changing climate and performance of dwellings should shed light on possible adaptation strategies since rural population tend to be proactive in responding to change. Research reveals increased dependence on electro-mechanical equipment for thermal comfort, clothing adjustments and window operation as some of the major adaptation strategies^41,42^. Future work should aim to explore adaptation and mitigation strategies adopted in form, materials, or surface treatments in the dwelling elements and in clothing, window operation and use of spaces by the occupants.
  However, understanding how people are going to change as climate warms, would involve intergenerational attitude and physiological studies, as current forecasts rely on thermal comfort responses valid for the current (adult) generation. Attitude is a critical determinant of sustainablity in human settlements and determines how the built environment evolves, with forcasting models relying on current generation attitudes^45^. In 2050, the present-day infants and youth would be the adults responsible for affecting changes or responses to climate change vulnerabilities. Given the diversity in ethnicity and other physiological differences, such studies on human exposure to climatic variations are extremely challenging in providing the causal basis required for forecasting^43,44^.

## References

[1] Global Alliance for Buildings and Construction, International Energy Agency & United Nations Environment Programme. *2019 Global status report for buildings and construction: Towards a zero-emissions, efficient and resilient buildings and construction sector*. (2019). ISBN No: 978-92-807-3768-4

[2] de Wilde P, Coley D (2012). The implications of a changing climate for buildings. Build. Environ..

[3] IEA. *Transition to sustainable buildings: Strategies and opportunities to 2050*. (2013). ISBN: 978-92-64-20241-2

[4] GEA. Chapter 10. Energy end-use Buildings, in *Global energy assessment toward a sustainable future* (eds. Johansson, T. B., Patwardhan, A., Nakicenovic, N. & Gomez-Echeverri, L.) 649–760 (Cambridge University Press and International Institute for Applied Systems Analysis, 2012). ISBN: 97805211829355

[5] US Energy Information Administration. *International Energy Outlook 2019 with projections to 2050*. (2019). www.eia.gov/ieo. Accessed on 22-04-2020

[6] The World Bank. *World Bank Open Data*. (2018). https://data.worldbank.org/indicator/SP.RUR.TOTL.ZS last accessed on 12-02-2021

[7] Anandan, M. & Sankaravelu, R. Energy Uses in India: A Case of Electricity. *Int. J. Res. Commer. IT Manag.* **3,** 27–33 (2013). ISSN 2231–57568

[8] Chandran KM, Balaji NC, Mani M (2015). Understanding transitions in a rural Indian building typology in the context of well-being. Curr. Sci..

[9] Laurie Baker Centre for Habitat Studies. *Housing conditions in India: With special focus on rural areas and socially disadvantaged sections*. **I,** (2014).

[10] Prayas (Energy Group). *Residential electricity consumption in India : What do we know?* (2016).

[11] Henna, K., Saifudeen, A. & Mani, M. Responsiveness and resilience of existing dwellings in warm-humid climate zone to changing climate. in *Proc. 1st Int. Conf. Comf. Extrem. Energy, Econ. Clim.* (ed. Finlayson, S. R. and W.) 758–773 (2019).

[12] Li DHW, Yang L, Lam JC (2012). Impact of climate change on energy use in the built environment in different climate zones—A review. Energy.

[13] Wang H, Chen Q (2014). Impact of climate change heating and cooling energy use in buildings in the United States. Energy Build..

[14] Karimpour M, Belusko M, Xing K, Boland J, Bruno F (2015). Impact of climate change on the design of energy efficient residential building envelopes. Energy Build..

[15] Huang J, Gurney KR (2016). The variation of climate change impact on building energy consumption to building type and spatiotemporal scale. Energy.

[16] Mani, M., Dayal, A. & Chattopadhyay, R. N. An Assessment into the sustainability of earthen structures and modern transitions, in *International symposium on earthen structures*, pp. 22–24 (2007).

[17] Singh MK, Mahapatra S, Atreya SK (2009). Bioclimatism and vernacular architecture of north-east India. Build. Environ..

[18] Shastry V, Mani M, Tenorio R (2016). Evaluating thermal comfort and building climatic response in warm-humid climates for vernacular dwellings in Suggenhalli (India). Archit. Sci. Rev..

[19] Praseeda KI, Mani M, Reddy BVV (2014). Assessing impact of material transition and thermal comfort models on embodied and operational energy in vernacular dwellings (India). Energy Procedia.

[20] Dili AS, Naseer MA, Varghese TZ (2010). Thermal comfort study of Kerala traditional residential buildings based on questionnaire survey among occupants of traditional and modern buildings. Energy Build..

[21] Shastry V, Mani M, Tenorio R (2014). Impacts of modern transitions on thermal Comfort in vernacular dwellings in warm-humid climate of Sugganahalli (India). Indoor Built Environ..

[22] Bureau of Indian Standards. *National Building Code of India 2005*. 883 (Bureau of Indian Standards, 2005).

[23] Brager GS, de Dear RJ (1998). Thermal adaptation in the built environment : a literature review. Energy Build..

[24] Crawley DB (2001). EnergyPlus: Creating a new-generation building energy simulation program. Energy Build..

[25] Meteotest. Meteonorm Handbook part I : Software version 7.3.4. (2020).

[26] IPCC. *Climate Change 2007: Synthesis Report. Contribution of Working groups I, II and III to the fourth assessment report of the intergovernmental panel on climate change*. [Core Writing Team, Pachauri, R.K. and Reisinger, A. (eds.)] (IPCC, Geneva, Switzerland, 2007). ISBN 92-9169-122-4

[27] Mani M, Reddy BVV (2012). Sustainability in human settlements: Imminent material and energy challenges for buildings in India. J. Indian Inst. Sci..

[28] Belz, M. M. Unconscious landscapes: Identifying with a changing vernacular in Kinnaur, Himachal Pradesh. *Mater. Cult.* **45,** 1–27 (2013). http://www.jstor.org/stable/24397619 Last accessed: 03-11-2017.

[29] ASHRAE. *Thermal Environmental Conditions for Human Occupancy*. (2010). ISSN 1041-2336

[30] de Dear RJ, Brager GS (1998). Developing an adaptive model of thermal comfort and preference. ASHRAE Trans..

[31] de Dear RJ, Brager GS (2002). Thermal comfort in naturally ventilated buildings : revisions to ASHRAE Standard 55. Energy Build..

[32] de Dear, R. Adaptive comfort applications in Australia and impacts on building energy consumption. in *IAQVEC 2007 Proceedings of the 6th international conference on indoor air quality, ventilation and energy conservation in buildings: sustainable built environment* **2,** 1–8 (2007).

[33] ASHRAE. *ASHRAE Guideline 14: Measurement of Energy and Demand Savings*. (2002). ISSN 1049-894X

[34] Coakley D, Raftery P, Keane M (2014). A review of methods to match building energy simulation models to measured data. Renew. Sustain. Energy Rev..

[35] Allesina G, Mussatti E, Ferrari F, Muscio A (2018). A calibration methodology for building dynamic models based on data collected through survey and billings. Energy Build..

[36] Royapoor M, Roskilly T (2015). Building model calibration using energy and environmental data. Energy Build..

[37] Carlander J, Trygg K, Moshfegh B (2019). Integration of measurements and time diaries as complementary measures to improve resolution of BES. Energies.

[38] Hoes P, Hensen JLM, Loomans MGLC, de Vries B, Bourgeois D (2009). User behavior in whole building simulation. Energy Build..

[39] Qiu S, Li Z, Pang Z, Zhang W, Li Z (2018). A quick auto-calibration approach based on normative energy models. Energy Build..

[40] Ruiz GR, Bandera CF (2017). Validation of calibrated energy models: Common errors. Energies.

[41] Bonte M, Thellier F, Lartigue B (2014). Impact of occupant’s actions on energy building performance and thermal sensation. Energy Build..

[42] Chen S (2020). Effect of inhabitant behavioral responses on adaptive thermal comfort under hot summer and cold winter climate in China. Build. Environ..

[43] Evans GW (2019). Projected behavioral impacts of global climate change. Annu. Rev. Psychol..

[44] Schweiker M, Huebner GM, Kingma BRM, Kramer R, Pallubinsky H (2018). Drivers of diversity in human thermal perception—A review for holistic comfort models. Temperature.

[45] Mani, M., Varghese, K. & Ganesh, L.S. Integrated model framework to simulate sustainability of human settlements. ASCE J. of Urban Planning and Development, **131**(3), 147–158 (2005)

